# Methylation-Mediated Silencing of MicroRNA-497 Promotes Breast Cancer Progression Through Up-Regulation of Mucin1

**DOI:** 10.3389/fonc.2020.552099

**Published:** 2020-10-23

**Authors:** Shuang Tao, Hong Li, Xiuzhen Ma, Bin Lian, Jiale He, Yali Gao, Jinping Li

**Affiliations:** ^1^Department of Breast Surgery, Changzhou No. 7 People’s Hospital, Changzhou, China; ^2^Department of Surgical Oncology, General Hospital of Ningxia Medical University, Yinchuan, China; ^3^Ningxia Medical University, Yinchuan, China

**Keywords:** MicroRNA-497, DNA methylation, mucin1, breast cancer, proliferation, migration, apoptosis

## Abstract

**Background:**

Potential anti-tumor effects of microRNA-497 (miR-497) have been highlighted in various malignancies including breast cancer. However, little is known about the function of miR-497 and its putative target mucin1 (MUC1) in breast cancer. The present study explored how miR-497 regulates breast cancer progression in a MUC1-dependent manner.

**Methods:**

Expression of miR-497 and MUC1 was determined in breast cancer tissues and cells. Methylation specific polymerase chain reaction was used to measure the methylation status of CpG islands of miR-497 promoter, while chromatin immunoprecipitation assay was used to detect recruitment of methyltransferase to the promoter region of miR-497. Alteration in expression of miR-497 (overexpression) and MUC1 (up- and down-regulation) was performed to examine their roles in breast cancer biology *in vitro* and *in vivo*. The binding affinity between miR-497 and MUC1 was investigated through a bioinformatics database and dual luciferase reporter gene assay.

**Results:**

MiR-497 was down-regulated and MUC1 was up-regulated in breast cancer tissues and cell lines. Besides, methylation induced a down-regulation of miR-497 in breast cancer. The bioinformatics analysis and dual luciferase reporter gene assay indicated that miR-497 targeted MUC1. Overexpression of miR-497 inhibited breast cancer cell proliferation and invasion and promoted the apoptosis of breast cancer cells by down-regulating MUC1. The inhibitory action of miR-497 on tumor growth was validated *in vivo.*

**Conclusion:**

In conclusion, miR-497 down-regulated MUC1 expression and subsequently suppressed breast cancer progression, highlighting miR-497 to be a potential biomarker and therapeutic target for breast cancer therapy.

## Introduction

Breast cancer is a malignant tumor that occurs in the epithelial tissues of the breast gland, which ranks first in morbidity and mortality among malignant tumors of women in China and many other countries (1). While the formerly poor prognosis of breast cancer has yielded to decades of study, there is still an urgent requirement to develop more effective and specific treatments for this disease (2).

MicroRNAs (miRNAs) are a class of non-coding single-stranded RNA of approximately 22 nucleotides in length encoded by endogenous genes, which function not only as important regulators of gene expression, but also play important roles in cancer progression (3, 4). According to a recent study, treatment with a histone deacetylase inhibitor decreases tumorigenesis and promotes intrinsic human breast cancer cell apoptosis by inducing the expression of miR-125a-5p *in vivo* and *in vitro* (5). Additionally, overexpression of miR-340 inhibits the migration and invasion of tumor cells, while knockdown of miR-340 promotes the migration and invasion of breast cancer cells (6). Furthermore, overexpression of microRNA-497 (miR-497) in human NPM-ALK-positive anaplastic large cell lymphoma cells suppresses cell proliferation *via* targeting cyclin-dependent kinase 6, E2F transcription factor 3, and cyclin E1 (7). In addition, up-regulation of structure specific recognition protein 1 favors the development of hepatocellular carcinoma, whereas miR-497 suppresses the progression of that tumor by negatively regulating its expression (8). In a previous study, miR-497 has been demonstrated to exert inhibitory effects on breast cancer malignancy (9).

Scrutiny of a bioinformatic database (starBase) predicted mucin1 (MUC1) as the target of miR-497. A recent study revealed that miR-206 inhibits the progression of gastric cancer by directly targeting MUC1 and reducing its expression in gastric cancer cells (10). Moreover, miR-1291 also targets MUC1 to accelerate the apoptosis of esophageal squamous cell carcinoma cells (11). MUC1 is a type I transmembrane protein, which is normally expressed in the proximal luminal or glandular surface of epithelial cells in various tissues and organs. Breast cancer tissues have remarkably high MUC1 expression compared to normal tissues (12). Additionally, clinical research has shown that overexpression of MUC1 imparts poor prognosis in patients with breast cancer (13).

Based on the above literature, we hypothesized that overexpression of miR-497 involves in the pathophysiological processes of breast cancer by regulating MUC1. Therefore, we explored the interaction between miR-497 and MUC1, aiming to identify a potential new strategy for breast cancer treatment.

## Materials and Methods

### Ethics Statement

The current study performed with the approval of the Ethics Committee of General Hospital of Ningxia Medical University. All patients recruited in the study signed informed consents documents. The animal experiments were approved by the Experimental Animal Ethics Committee of General Hospital of Ningxia Medical University.

### Study Subjects

Breast cancer tissues and normal adjacent tissues (>3 cm from cancer tissues) were collected from 68 female patients with breast cancer admitted to General Hospital of Ningxia Medical University from January 2006 to January 2008. The ages of patients were of mean age of 51.10 ± 6.22 years (range 39 to 70). The diagnosis of invasive breast cancer was made by surgical pathological examination following pre-surgical magnetic resonance-diffusion weighted imaging (MR-DWI) of the breast. Patients who met the following criteria were excluded from this study: (1) Patients without complete clinical record; (2) Patients with a history of breast augmentation, mastitis, radiotherapy, chemotherapy, and breast-related surgery; (3) Patients treated during pregnancy or lactation; (4) The interval between MR-DWI and surgery exceeded more than 1 month; (5) Recurrence and/or distant metastasis occurred after treatment; and (6) Patients with mental disorders who could not complete the questionnaire unassisted.

### Cell Culture and Transfection

Breast cancer cell lines MCF-7, MDA-MB-468, MDA-MB-453, and MDA-MB-231 and normal breast epithelial cell line MCF-10A were purchased from ATCC (Manassas, VA, United States). The cell lines were cultured in Dulbecco Modified Eagle Medium (DMEM) containing 10% fetal bovine serum (FBS; Gibco, Grand Island, NY, United States), 100 U/mL penicillin, and 100 mg/mL streptomycin. Cells were seeded into 6-well plates and cultured for 24 h at 37°C to reach 75% confluence. Cells were then transfected with short hairpin RNA (shRNA) targeting MUC1 (sh-MUC1), plasmids overexpressing MUC1 (oe-MUC1), miR-497 mimic, miR-497 agomir (Agomir is a specially labeled and chemically modified double-stranded small RNA, which regulates the biological functions of target genes by simulating endogenous miRNA) or their negative controls (sh-NC, oe-NC, mimic-NC, and NC agomir) using the Lipofectamine 2000 kit (Invitrogen, Carlsbad, CA, United States). All plasmids were constructed by and purchased from Shanghai GenePharma Co., Ltd., (Shanghai, China) and were used at a concentration of 50 ng/mL. Cells were treated with DNA methyltransferase inhibitor 5-aza-2′-deoxycytidine (5-aza-dc) or dimethyl sulfoxide (DMSO) as vehicle control (Sigma-Aldrich, St Louis, MO, United States).

### Reverse Transcription-Quantitative Polymerase Chain Reaction

Total RNA was extracted using Trizol reagent (15596026, Invitrogen) and RT was performed using the NCode^TM^ miRNA First-Strand complementary DNA (cDNA) Synthesis Kit (Thermo Fisher Scientific Inc., Waltham, MA, United States). The synthesized cDNA was subjected to reverse transcription-quantitative polymerase chain reaction (RT-qPCR) detection using a Fast SYBR Green PCR Kit (Applied Biosystems, Carlsbad, CA, United States) in an ABI PRISM 7300 RT-qPCR System instrument (Applied Biosystems). Each reaction was conducted in triplicate. The relative expression of miR-497 (normalized to U6) and MUC1 [normalized to glyceraldehyde-3-phosphate dehydrogenase (GAPDH)] was calculated using the 2^–ΔΔCT^ method. Primers are shown in Table 1.

**TABLE 1:** Primer sequences for reverse transcription quantitative polymerase chain reaction.

Gene	Sequence (5′-3′)
miR-497	Forward: CGCCAGCAGCACACTGTGG
	Reverse: GTGCAGGGTCCGAGGT
U6	Forward: CTCGCTTCGGCAGCACA
	Reverse: AACGCTTCACGAATTTGCGT

*miR-497, microRNA-497.*

### Methylation Specific PCR

The methylation status of the miR-495 promoter region was detected using the DNA Methylation-Gold^TM^ Kit (D5005, Zymo Research, Irvine, CA, United States). In brief, 900 μL water, 50 μL M-Dissolving Buffer and 300 μL M-Dilution Buffer were added into one tube of CT Conversion Reagent and mixed for 10 min. Then, a 20 μL DNA sample was supplemented with 130 μL of this prepared CT Conversion Reagent, followed by treatment at 98°C for 10 min, at 64°C for 2.5 h, and stored at 4°C for further use. Subsequently, 600 μL M-Binding Buffer was added into a Zymo-Spin IC Column and mixed by inverting. Centrifugation followed at >10,000 *g* for 30 s. After addition of 100 μL M-Wash Buffer into the column, centrifugation was repeated for another 30 s, followed by addition of 200 μL M-desulphonation buffer. The sample was allowed to stand for 15–20 min. After another round of centrifugation for 30 s, 200 μL M-Wash Buffer was added into the column, which was then centrifuged for 30 s. The column matrix was added with 10 μL M-Elution Buffer, placed into a 1.5 mL Eppendorf tube and centrifuged for elution of the DNA. The methylation reaction primer sequences used for methylation specific PCR (MSP) amplification were miR-497-MF (5′-ATAAGGACGGGGATATATATATCGT-3′) and miR-497-MR (5′-AAACTACTTCCTTTACCTAAAACGC-3′) with an amplification length of 220 bp. The primer sequences used for the unmethylation reaction were miR-497-UF (5′-ATAAGGATGGGGATATATATATTGT-3′) and miR-497-UR (5′-AACTACTTCCTTTACCTAAAACACC-3′) with an amplification length of 205 bp. The purified DNA was modified with bisulfite, purified, and subjected to PCR reaction. The reaction product was subjected to agarose gel electrophoresis and photograph, followed by analysis in an image analysis system.

### Chromatin Immunoprecipitation Assay

Cells on a 10 cm plate was added with 243 μL 37% formaldehyde (final concentration: 1%) and incubated at 37°C for 10 min. Crosslinking was terminated by addition of 450 μL 2.5 M glycine to a final concentration of 0.125 M. The sample was then allowed to stand at room temperature for 5 min. With the removal of the medium, cells were washed with cold phosphate buffer saline (PBS) twice and then collected into a 15 mL centrifuge tube. Centrifugation (2000 rpm for 5 min) was conducted for collection of cells. After discarding the supernatant, sodium dodecyl sulfate (SDS) lysis buffer was added until the final concentration was 1 × 10^6^ cells/100 μL. Protease inhibitor was added and cells were disrupted by ultrasound, followed by centrifugation at 10,000 × *g* for 10 min at 4°C. With the removal of insoluble substance, 300 μL sample was used for subsequent experiments while the remaining sample was stored at −80°C for later use. The sample was added 100 μL of a solution containing antibodies to DNA methyltransferase 1 (DNMT1, 1:100, ab13537, Abcam, Cambridge, MA, United States), DNMT3a (1:100, ab2850, Abcam), and DNMT3b (1:50, ab2851, Abcam) for binding with miR-497 promoter as the experimental group. Another 100 μL portion remained untreated as the control group, and another 100 μL sample was added with 4 μL 5 M NaCl (final concentration: 0.2 M) for de-crosslinking at 65°C for 3 h. Electrophoresis was performed to detect the efficiency of ultrasonic cell disruption. Then, 900 μL Chromatin Immunoprecipitation (ChIP) Dilution Buffer and 20 μL 50 × PIC were added into a 100 μL portion disrupted products, followed by addition of 60 μL ProteinA Agarose/Salmon Sperm DNA for 1-h incubation at 4°C. The sample was then precipitated at 4°C for 10 min. The supernatant was collected through centrifugation at 700 rpm for 1 min. Then, 20 μL sample was reserved as input. One tube was added with 1 μL antibody while the other tube remained untreated for incubation at 4°C overnight. Subsequently, 100 μL disrupted products were de-crosslinked by addition of 4 μL 5 M NaCl at 65°C for 2 h. Each tube was added with 60 μL ProteinA and Agarose/Salmon Sperm DNA and incubated at 4°C for 2 h. The sample was allowed to stand at 4°C for 10 min and centrifuged at 700 rpm for 1 min. The supernatant was removed and the precipitate complex was washed over inverting at 4°C for 10 min and then left to stand at 4°C for 10 min followed by centrifugation at 700 rpm for 1 min. With the removal of the supernatant, elution buffer was prepared by mixing 100 μL 10% SDS, 100 μL 1 M NaHCO_3_, and 800 μL ddH_2_O (total volume: 1 mL). A 250 μL portion elution buffer was added into each tube, which was then mixed by inversion for 15 min at room temperature. The supernatant was harvested through centrifugation. Finally, the volume of elution buffer was 500 μL in each tube, which was added and mixed with 20 μL 5 M NaCl (final concentration: 0.2 M), followed by de-crosslinking at 65°C overnight. Each tube was then supplemented with 1 μL RNaseA (MBI, Lithuania) for 1-h incubation at 37°C, and then treated with 10 μL 0.5 M ethylenediaminetetraacetic acid, 20 μL 1 M Tris–HCl (pH = 6.5) and 2 μL 10 mg/mL protease K at 45°C for 2 h. The test kit was retrieved using omega gel. At last, the sample was dissolved in 100 μL ddH_2_O, and the enrichment of DNA fragments in the miR-497 promoter was detected by qPCR. The length of used primer sequence was 125 bp.

### Western Blot Analysis

The cultured cells were lysed by enhanced radio immunoprecipitation assay (RIPA) lysis (Wuhan Boster Biological Technology Co., Ltd., Wuhan, China) containing a protease inhibitor (1 mM phenylmethylsulfonyl fluoride), and the protein concentration was determined using a Bicinchoninic Acid Protein Quantification Kit (Wuhan Boster Biological Technology). The protein was separated by 10% SDS-polyacrylamide gel electrophoresis and transferred to a polyvinylidene fluoride membrane. For immunoblotting, the membrane was incubated in rabbit primary antibodies, i.e., anti-MUC1 (1:1000, ab45167, Abcam), anti-proliferating cell nuclear antigen (PCNA; 1:2000, ab18197, Abcam), anti-Ki67 (1:2000, ab16667, Abcam), anti-B-cell lymphoma-2 (Bcl-2)-associated protein X (Bax; 1:5000, ab32503, Abcam), and anti-cleaved-caspase 3 (1:500, ab13847, Abcam). The secondary antibodies included: horseradish peroxidase (HRP)-conjugated goat anti-mouse immunoglobulin G (IgG; 1:2000, ab205719, Abcam) and HRP-conjugated rabbit anti-mouse IgG (1:1000, ab6728, Abcam). The membrane was developed with enhanced chemiluminescence luminescent solution (Merck Millipore, Billerica, MA, United States) and imaged. The ImageJ software developed by National Institutes of Health was used for densitometry analysis, with GAPDH used as internal reference.

For western blot analysis, cancer and normal adjacent tissues were, respectively, rinsed by pre-cooled PBS, mixed in RIPA buffer, and sonicated for 5–8 min. The supernatant was collected through centrifugation and loaded for the subsequent western blot analysis.

### 5-Ethynyl-2′-Deoxyuridine Incorporation Assay

Cells cultured in 24-well plate were incubated with 10 μM 5-Ethynyl-2′-Deoxyuridine (EdU; C1034-1, Guangzhou Ribo Biotechnology Co., Ltd., Guangzhou, Guangdong, China) for 2 h, followed by fixation with 4% paraformaldehyde for 15 min. The cells were then permeabilized with 0.5% Triton X-100 in PBS for 20 min. Afterward, the cells were stained with 100 μL of the staining solution. Nuclei were stained with 4′6-diamidino-2-phenylindole (DAPI) for 5 min. The cells from 6–10 randomly selected fields were observed under a fluorescence microscope and the density of EdU labeled cells was recorded and expressed as the percentage of EdU labeled cells: EdU labeling rate = EdU labeled cells/(EdU labeled cells + EdU unlabeled cells).

### Transwell Invasion Assay

The *in vitro* invasion assay was performed as previously described (14) using Transwell inserts with 8.0 μm pore size. In brief, cells starved for 12 h were suspended in serum-free medium at the density of 1 × 10^5^ cells/mL. Then, 100 μL portions of cell suspension were seeded onto the transwell inserts pre-coated with Matrigel (Becton, Dickinson and Company, NJ, United States). The transwell inserts were then placed into 24-well plate containing complete culture medium. After 24-h incubation, the upper surface of the transwell inserts was wiped with a cotton swab. The invaded cells were fixed with 100% methanol and stained with 1% toluidine blue (Sigma-Aldrich). The number of invaded cells was recorded under an inverted microscope (Carl Zeiss, Germany) in five randomly-selected fields per well.

### Flow Cytometry

Cells were centrifuged (2,000 rpm, 5 min) and washed twice with ice-cold PBS. The cells were then suspended with 400 μL of 1 × Binding Buffer, and incubated with 5 μL of Annexin V-fluorescein isothiocyanate at 4°C for 15 min in the dark. Next, 10 μL of propidium iodide was added to the cell suspension and incubated at 4°C for 5 min in the dark. Cell apoptosis was measured by flow cytometer instrument (BD FACS Calibur, Becton, Dickinson and Company, NJ, United States) within 1 h of the reaction.

### Dual-Luciferase Reporter Gene Assay

Target gene analysis of miR-497 and MUC1 was performed using a biological prediction website. The 3′-untranslated region (3′-UTR) of the MUC1 mRNA sequence containing the predicted (wild type) or mutated binding sites for miR-497 was cloned into luciferase reporter vectors. Cells were co-transfected with these vectors and miR-497 mimic or mimic-NC and cultured for 48 h. After successful transfection, the cells were cultured for 48 h and then collected. Changes in the dual luciferase activity of miR-497 and MUC1 in cells were determined following instruction in the Dual Luciferase Assay Kit (Genecopoeia, D0010, Beijing Solarbio Science & Technology Co., Ltd., Beijing, China). Fluorescence intensity was measured using a Promega Glomax 20/20 luminometer (Shaanxi Zhongmei Biotechnology Co., Ltd., Shaanxi, China).

### Xenografts in Nude Mice

Forty healthy nude mice (Institute of Materia Medica Chinese Academy of Medical Sciences, Beijing, China; 4–6 weeks old, weighing 18–22 *g* with a mean of 19.85 ± 1.35 *g*) were raised in SPF animal laboratory cages. The laboratory was held with relative humidity of 60 to 65% and temperature of 22 to 25°C, with free access to food and water under a 12-h light and dark cycle. The experiment was started after 1 week of adaptive feeding, and the health status of the nude mice was observed before the experiment. These mice were randomly assigned into four groups and received subcutaneous injection of cell suspension (1 × 10^7^ cells/mL) treated with sh-NC, sh-MUC1, agomir-NC, or miR-497 agomir, respectively. 5 weeks later, the mice were euthanized and tumor volume and weight were measured.

### Statistical Analysis

Statistical analysis was performed using SPSS 21.0 software (IBM, Armonk, NY, United States). The measurement data were expressed as mean ± standard deviation. The normality and variance homogeneity tests were performed. The paired *t*-test was used for comparison of data within groups with normal distribution and equal variance, and unpaired *t*-test for data comparison between two groups. Data among multiple groups were analyzed by one-way analysis of variance (ANOVA), followed by Tukey’s *post hoc* test. Data between two groups at different time points were compared by repeated measures ANOVA. When the data did not show normal distribution or equal variance, the rank-sum test was conducted. A value of *p* < 0.05 was considered to be statistically significant.

## Results

### Methylation Down-Regulates miR-497 Expression in Breast Cancer

**FIGURE 1:**
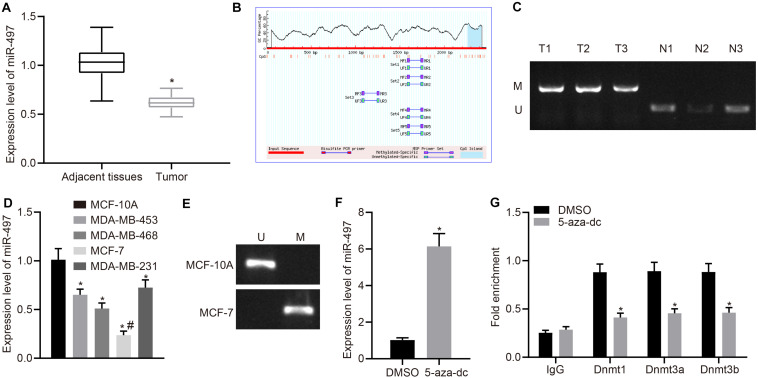
Methylation induced miR-497 down-regulation in breast cancer. **(A)** Expression of miR-497 in breast cancer tissues and adjacent normal tissue was determined by RT-qPCR (*n* = 68), *p < 0.05 compared with the normal adjacent tissues. **(B)** MethPrimer was used to predict the presence of CpG islands upstream of miR-497 promoter. **(C)** MSP was used to measure promoter methylation status in breast cancer tissues and adjacent normal tissues. **(D)** RT-qPCR was used to determine expression of miR-497 in breast cancer cells and normal breast epithelial cells, *p < 0.05 compared with MCF-10A cell line, #*p* < 0.05 compared with MDA-MB-453, MDA-MB-468, and MDA-MB-231 cell lines. **(E)** MSP was used to measure promoter methylation status in breast cancer cells and normal breast epithelial cells. **(F)** Relative expression of miR-497 in MCF-7 treated with or without 5-aza-dc, *p < 0.05 compared with DMSO. **(G)** ChIP assay was used to measure the enrichment of DNMT1/DNMT3 (a/b) to the promoter region of miR-497, *p < 0.05 compared with DMSO. The measurement data were expressed as mean ± standard deviation. Data between two groups were analyzed by unpaired *t*-test, and data between cancer tissues and adjacent tissues were analyzed by paired *t*-test. Data among multiple groups were analyzed by one-way ANOVA, followed by Tukey’s *post hoc* test. Cell experiment was repeated three times independently.

To investigate expression of miR-497 in breast cancer, we performed RT-qPCR in human surgical samples and cell lines. The results indicated that miR-497 expression was down-regulated in breast cancer tissues compared with that in the normal adjacent tissues (*p* < 0.05; Figure 1A). Similar results were observed in cell lines, in which the miR-497 was down-regulated in breast cancer cell lines (MDA-MB-453, MDA-MB-468, MCF-7, and MDA-MB-231) compared with that in a normal breast epithelial cell line (MCF-10; Figure 1D). MCF-7, in which the miR-497 expression was the lowest, was selected for further experiments. Consistent with present results, a previous study has revealed that hypermethylation of CpG islands upstream of miR-497 results in its decreased expression in breast cancer (9). Using online database^1^, we predicted the potential CpG island for miR-497 (Figure 1B) and designed primers for MSP specific primers to confirm our prediction. The methylation status of the predicted CpG island was measured in breast cancer tissues by MSP. We observed the methylation of the CpG island in breast cancer tissues but not in normal adjacent tissues (Figure 1C). MSP was also performed using normal epithelial cell line and breast cancer cell line, yielding similar results. In MCF-10A cells, the CpG island in the miR-497 promoter was not methylated, but in MCF-7 cells, the methylation could be detected (Figure 1E). Moreover, treatment with a DNA methyltransferase inhibitor (5-Aza-dc) resulted in up-regulation of miR-497 in MCF-7 cells (Figure 1F), which further confirmed that miR-497 expression was under regulation by DNA methylation. Furthermore, exposure to 5-Aza-dc also led to reduced recruitment of DNA methyltransferase (DNMT1, DNMT3a, and DNMT3b) to the promoter region of miR-497 (Figure 1G). These results suggested that miR-497 was down-regulated in breast cancer due to the hypermethylation at its promoter CpG island.

### Restored miR-497 Inhibits Breast Cancer Cell Malignant Phenotypes

**FIGURE 2:**
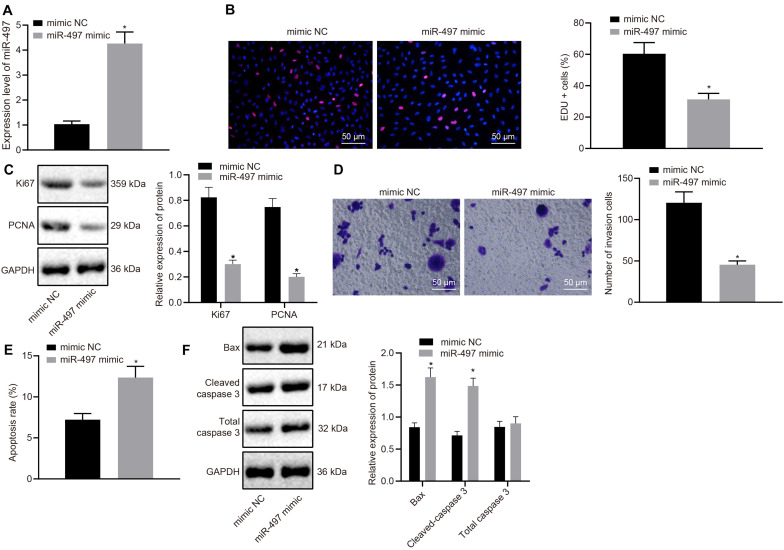
Up-regulated miR-497 inhibited breast cancer cell proliferation, and invasion and promoted apoptosis. **(A)** The overexpression of miR-497 was confirmed in breast cancer cells after 24 h transfection of miR-497 mimic by RT-qPCR. **(B)** The proliferation of breast cancer cells after 36 h transfection of miR-497 mimic was measured using the EdU incorporation assay. **(C)** Expression Ki67 and PCNA, normalized to GAPDH, in MCF-7 cells after 48 h transfection of miR-497 mimic was measured using western blot analysis. **(D)** The invasion of MCF-7 cells after 24 h transfection of miR-497 mimic was measured using Transwell invasion assay. **(E)** The apoptosis of MCF-7 cells after 48 h transfection of miR-497 mimic was confirmed by flow cytometry. **(F)** Expression Bax, total caspase 3 and cleaved-caspase 3, normalized to GAPDH, in MCF-7 cells after 48 h transfection of miR-497 mimic was measured using western blot analysis. *p < 0.05 compared with the cells transfected with mimic-NC. The measurement data were expressed as mean ± standard deviation, and data between two groups were analyzed by unpaired *t*-test. Cell experiments were repeated three times independently.

To investigate the effect of miR-497 on the biological function of breast cancer *in vitro*, miR-497 was overexpressed in MCF-7 cells. MCF-7 cells were transfected with miR-497 mimic and the up-regulation was confirmed by RT-qPCR (Figure 2A). Cell proliferation was measured using EdU incorporation assay and miR-497 mimic reduced the proliferation of MCF-7 cells compared with that in the control group, in which MCF-7 cells were transfected with mimic-NC (Figure 2B). Expression of proliferation-related proteins (Ki67 and PCNA) was measured using western blot analysis. Expression of Ki67 and PCNA was decreased in the cells transfected with miR-497 mimic compared with that in the cells transfected with mimic-NC (*p* < 0.05; Figure 2C).

A transwell invasion assay was performed to determine the role of miR-497 in breast cancer invasion. We observed that, compared with MCF-7 cells transfected with mimic-NC, MCF-7 cells overexpressing miR-497 displayed reduced invasive potency (Figure 2D). We further investigated the role of miR-497 in breast cancer cell apoptosis by flow cytometry. The abundance of apoptotic breast cancer cells was higher after transfecting with miR-497 mimic than in the cells transfected with mimic-NC (*p* < 0.05; Figure 2E). Results from western blot analysis also showed that expression of apoptosis-related proteins (Bax and cleaved-caspase 3) was up-regulated by miR-497 overexpression (Figure 2F). The above results suggested that up-regulated miR-497 functioned as a tumor suppressor in breast cancer by inhibiting the proliferation and invasion as well as promoting the apoptosis of the cancer cells.

### miR-497 Targets and Down-Regulates MUC1 Expression

**FIGURE 3:**
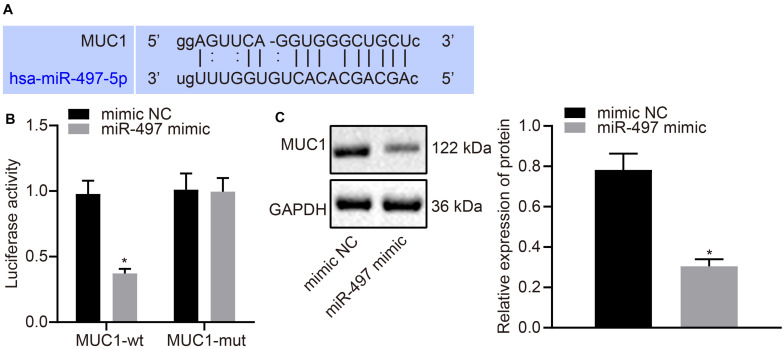
MUC1 can be targeted and down-regulated by miR-497 in breast cancer cells. **(A)** The predicted miR-497 binding site of MUC1. **(B)** Dual luciferase reporter gene assay was used to confirm the binding between miR-497 and MUC1 3′-UTR. **(C)** Expression of MUC1 normalized to GAPDH in breast cancer cells after 48 h transfection of miR-497 mimic was measured using Western blot analysis. *p < 0.05 compared with the cells transfected with mimic-NC. The measurement data were expressed as mean ± standard deviation, and data between two groups were analyzed by unpaired *t*-test. Cell experiments were repeated three times independently.

A bioinformatic prediction program (starBase) was used to predict the targets of miR-497, which identified MUC1 as a preferred target with a highly conserved binding sequence (Figure 3A). To confirm this result, dual luciferase reporter gene assay was performed in MCF-7 cells expressing reporter vector containing wild type MUC1 3′-UTR. The results showed that the luciferase activity was lower in cells co-transfected with miR-497 mimic than in cells co-transfected with mimic-NC (Figure 3B). On the other hand, when the mutated 3′-UTR of MUC1 was cloned into the reporter vector, the luciferase activity was unaffected by miR-497 overexpression (Figure 3B). These results suggested that miR-497 targeted MUC1 and the target was located at its 3′-UTR. MUC1 expression was measured using western blot analysis. MUC1 expression was reduced in the cells transfected with miR-497 mimic compared with that in the cells transfected with mimic-NC (*p* < 0.05; Figure 3C). The above results suggested that miR-497 could target MUC1 and down-regulate its expression in breast cancer cells.

### MUC1 Is Highly Expressed in Breast Cancer Tissues

**FIGURE 4:**
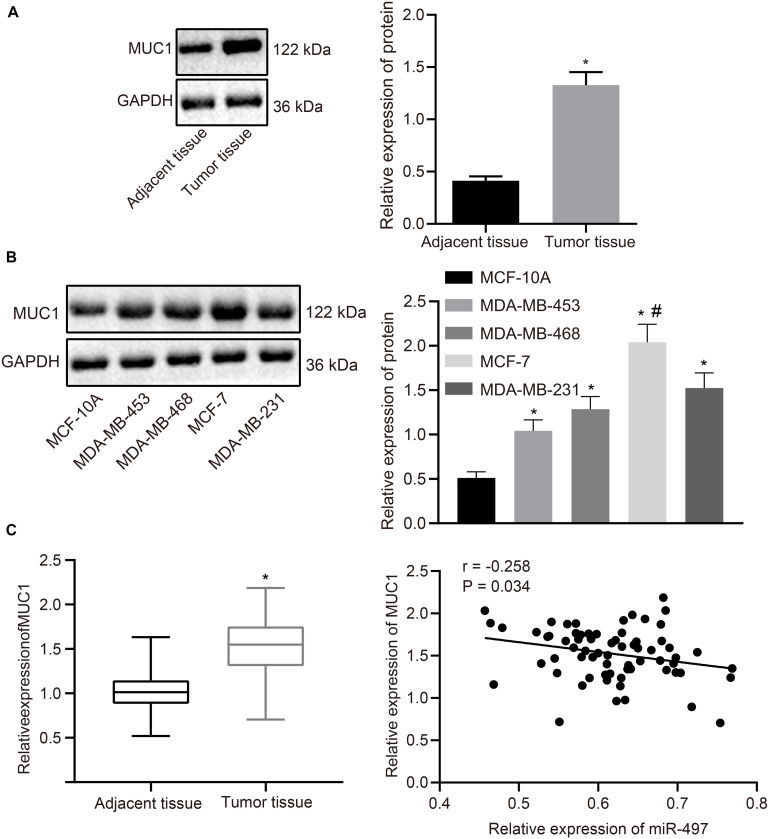
MUC1 is highly expressed in breast cancer tissues. **(A)** The relative expression of MUC1 normalized to GAPDH in breast cancer tissues and adjacent normal tissues determined by western blot analysis (*n* = 68). **(B)** The relative expression of MUC1 normalized to GAPDH in breast cancer cells and normal breast epithelial cells determined by western blot analysis. **(C)** The expression of MUC1 in breast cancer tissues and normal adjacent tissues (*n* = 68) determined by RT-qPCR, and Pearson correlation of MUC1 expression with miR-497 expression. *p < 0.05 compared with the normal adjacent tissues or MCF-10A cells. #*p* < 0.05 compared with MDA-MB-453, MDA-MB-468, and MDA-MB-231 cells. The measurement data were expressed as mean ± standard deviation, and data between cancer tissue and adjacent tissues were analyzed by paired *t*-test. Data among multiple groups were analyzed by one-way ANOVA, followed by Tukey’s *post hoc* test. Cells experiment were repeated three times independently.

Expression of MUC1 was determined in breast cancer tissues using western blot analysis. MUC1 was highly expressed in breast cancer tissues compared with the normal adjacent tissues (*p* < 0.05; Figure 4A). MUC1 expression in breast cancer cells was also measured using western blot analysis. Expression of MUC1 was increased in breast cancer cells (MDA-MB-453, MDA-MB-468, MCF-7, and MDA-MB-231) compared with that in normal epithelial cells (MCF-10A), where expression of MUC1 was highest in MCF-7 (Figure 4B). RT-qPCR results revealed significantly higher levels of MUC1 in breast cancer tissues than in normal adjacent tissues, and Pearson’s correlation coefficient suggested that MUC1 was negatively correlated with miR-497 (Figure 4C). The above results suggested that MUC1 was highly expressed in breast cancer cells.

### Restored MiR-497 Inhibits Breast Cancer Cell Malignant Phenotypes by Down-Regulating MUC1 Expression

**FIGURE 5:**
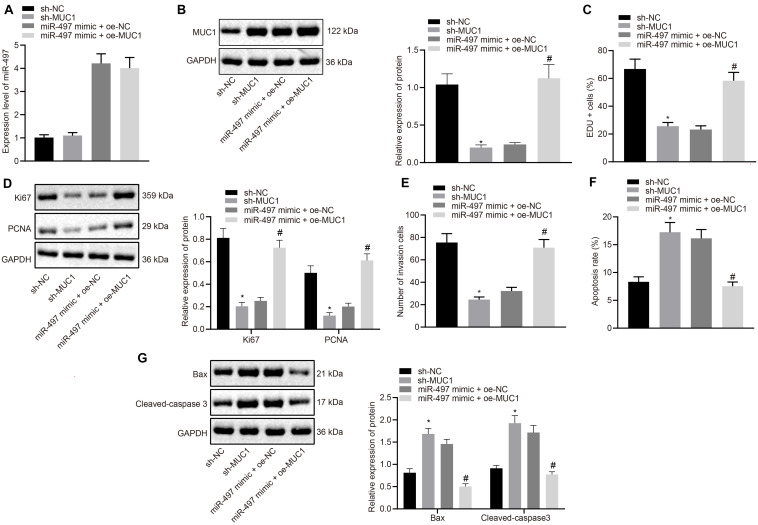
Up-regulated miR-497 inhibited the proliferation, and invasion, and promoted apoptosis of breast cancer cells by down-regulating MUC1. **(A)** Expression of miR-497 in MCF-7 cells after 24 h transfection was determined by RT-qPCR. **(B)** Expression of MUC1 normalized to GAPDH in MCF-7 cells after 48 h transfection was measured using western blot analysis. **(C)** The proliferation of MCF-7 cells after 36 h transfection was measured using EdU incorporation assay. **(D)** Expression of Ki67 and PCNA normalized to GAPDH in MCF-7 cells after 48 h transfection was measured using Western blot analysis. **(E)** The invasion potency of MCF-7 cells after 24 h transfection was measured using Transwell invasion assay. **(F)** The apoptosis of MCF-7 cells after 48 h transfection was confirmed by flow cytometry. **(G)** Expression of apoptosis-related factors Bax and cleaved-caspase 3 normalized to GAPDH in MCF-7 cells after 48 h transfection was measured using western blot analysis. *p < 0.05 compared with the cells transfected with sh-NC, #*p* < 0.05 compared with the cells co-transfected with miR-497 mimic + oe-NC. The measurement data were expressed as mean ± standard deviation, and data between two groups were analyzed by unpaired *t*-test. Cell experiments were repeated three times independently.

Our data has suggested that miR-497 negatively regulates MUC1 in breast cancer cells. So, we could hypothesize that the miR-497/MUC1 axis may play an important role in breast cancer progression. To test this hypothesis, we investigated the role of the miR-497/MUC1 axis in breast cancer progression *in vitro* using MCF-7 cells. First, we manipulated expression of miR-497 and/or MUC1 in MCF-7 cells by overexpressing miR-497, knocking down MUC1, or co-overexpressing miR-497 and MUC1. The results were analyzed by RT-qPCR and immunoblotting (Figures 5A,B).

Cell proliferation was measured using EdU incorporation assay. Compared with the control group (sh-NC), MUC1 knockdown reduced the proliferation of MCF-7 cells. Meanwhile, overexpressing miR-497 also resulted in comparably reduced proliferative potency of MCF-7 cells, although, further oe-MUC1 in those cells abolished the inhibitory effect achieved by miR-497 overexpression in isolation (Figure 5C). Moreover, results from western blot analysis of Ki67 and PCNA expression in those cells confirmed our findings from the proliferation assay *in vitro*. We observed that overexpressing miR-497 or knockdown of MUC1 resulted in decreased expression of Ki67 and PCNA. However, oe-MUC1 in MCF-7 cells overexpressing miR-497 restored the expression of MUC1 (Figure 5D).

Cell invasion determined by Transwell assay was reduced in MCF-7 cells transfected with sh-MUC1 compared with MCF-7 cells transfected with sh-NC (*p* < 0.05), and increased in MCF-7 cells co-transfected with miR-497 mimic and oe-MUC1 compared with MCF-7 cells co-transfected with miR-497 mimic and oe-NC (*p* < 0.05; Figure 5E).

Flow cytometry showed increased apoptosis of MCF-7 cells transfected with sh-MUC1 compared with MCF-7 cells transfected with sh-NC (*p* < 0.05), and decreased apoptosis of MCF-7 cells co-transfected with miR-497 mimic and oe-MUC1 compared with MCF-7 cells co-transfected with miR-497 mimic and oe-NC (*p* < 0.05; Figure 5F). Western blot showed increased expression of Bax and cleaved-caspase 3 in MCF-7 cells transfected with sh-MUC1 compared with MCF-7 cells transfected with sh-NC (*p* < 0.05), and showed reduced expression in MCF-7 cells co-transfected with miR-497 mimic and oe-MUC1 compared with MCF-7 cells co-transfected with miR-497 mimic and oe-NC (*p* < 0.05; Figure 5G). The above results suggested that up-regulated miR-497 inhibited breast cancer cell proliferation and promoted apoptosis by down-regulating MUC1.

### Restored miR-497 Inhibits Breast Cancer Growth *in vivo* by Down-Regulating MUC1 Expression

**FIGURE 6:**
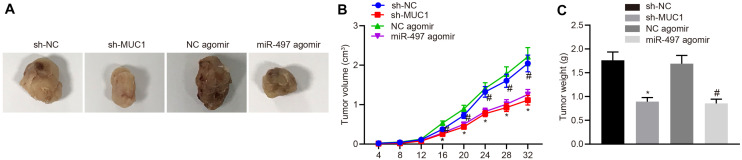
Up-regulated miR-497 inhibited breast cancer growth *in vivo* by down-regulating MUC1 expression. **(A)** Representative images of tumors from nude mice in each group. **(B)** Tumor growth curve of nude mice in each group. **(C)** Tumor weight of nude mice in each group. *p < 0.05 compared with mice treated with sh-NC, #*p* < 0.05 compared with mice treated with agomir-NC *n* = 10. The measurement data were expressed as mean ± standard deviation, and data between two groups were analyzed by unpaired *t*-test. Data among multiple groups at different time points were compared by repeated measures ANOVA, followed by Tukey’s *post hoc* test.

To study the effect of miR-497 and MUC1 on the growth of breast cancer *in vivo*, we monitored the size and weight of tumor formation in nude mice under various treatments. Results showed a reduction of tumors growth in the mice treated with sh-MUC1 compared with that with sh-NC, and there was a reduction of tumor growth in mice treated with miR-497 agomir compared with those treated with agomir-NC (*p* < 0.05; Figures 6A–C). Thus, up-regulated miR-497 inhibited breast cancer growth *in vivo* by down-regulating MUC1 expression.

## Discussion

As the most common tumor among females, breast cancer features a rather poor prognosis despite optimal treatment, and the lack of effective biomarkers hinders progress in improving outcomes (15). Notably, miRNAs have been reported to function as regulators of gene expression, affecting the development of many human diseases including cancers (16, 17). Of particular interest in the present context, miR-497 has been recognized to regulate the proliferation, invasion and survival of breast cancer cells (18). However, the underlying mechanism of these effects has not been comprehensively investigated until now. In the present study, we attempted to investigate the functional role of methylation-mediated miR-497 down-regulation in breast cancer progression. Collectively, results of our investigation showed that restored miR-497 could serve as a tumor suppressor in breast cancer by down-regulating expression of MUC1.

A key finding of the present study revealed that hypermethylation of the CpG island at the promoter region of miR-497 leads to its down-regulation in breast cancer cells and tissues. DNA methylation has been highlighted to be indicative of oncologic outcomes of patients with breast cancer (19). Likewise, miR-874 expression was reportedly diminished by DNA methylation in breast cancer, acting as a diagnostic and prognostic biomarker (20). This is also in alignment with other previous findings showing that DNA methylation down-regulates expression of miR-10a in gastric cancer cells (21) and mediates down-regulation of miR-449c in osteosarcoma cells (22). Furthermore, we found that miR-497 reduced the expression of proliferation-related factors Ki67 and PCNA, and up-regulated expression of apoptosis-related factors Bax and cleaved-caspase3 *in vitro*. Accumulating evidence suggests that the Ki67 index constitutes prognostic biomarker to estimate the proliferative activity of breast cancer tissues (23). Furthermore, overexpressed PCNA promotes cancer cell growth, colony formation and tumorigenesis of lung cancer cells, and suppresses the apoptosis of cancer cells (24). Another relevant finding is that the griffipavixanthone inhibits Bcl-2 expression and accelerates the apoptosis of human breast cancer cell MCF-7 *via* up-regulating expression of p53 and Bax (25). In a study investigating breast cancer, Moghtaderi et al. reported that the combination of the polysaccharide arabinogalactan and curcumin noticeably reduced cancer cell growth to promote the apoptosis of human breast cancer cells, and observably up-regulated the Bax/Bcl-2 ratio and expression of cleaved-caspase3 in MDA-MB-231 human breast cancer cells (26). Accordingly, we further confirmed that overexpression of miR-497 inhibited the proliferation and invasion of breast cancer cells and promoted their apoptosis. As in the present study, Zhong and his team also found reduced miR-497 expression in breast cancer tissues and cells compared with adjacent normal tissues and normal cells, and that low expression of miR-497 was associated with poor prognosis in the patients (27). Moreover, a previous study demonstrated that miR-497 inhibited tumorigenicity and angiogenesis in breast cancer cells and could thus be a potential target for breast cancer treatment (28).

We also observed in this study that MUC1, a key target of miR-497, was highly expressed in breast cancer cells, and that overexpression of miR-497 inhibited breast cancer cell proliferation and promoted apoptosis by down-regulating MUC1, thereby inhibiting breast cancer cell growth *in vivo*. A detailed examination by Shen et al. showed that miR-497 promoted the apoptosis of breast cancer cells *via* targeting Bcl-2 (29). Also, according to the findings by Luo et al., miR-497 may be a tumor suppressor gene for breast cancer, and overexpression of miR-497 can inhibit the proliferation, migration and invasion of breast cancer cells *via* targeting cyclin E1 (30). Moreover, there are a number of the direct targets of miR-497 in breast cancer, which involve genomic imprinting and histone modification in addition to DNA methylation (31), suggesting the need of further study of these various pathways. MUC1 is highly expressed in various cancer cells especially in breast cancer cells, and there is evidence indicating that the application of antibodies to MUC1 in combination with dinuclear platinum (II) complex can improve breast cancer treatment response (32). Moreover, our in our study we also silenced MUC1 to investigate its roles in breast cancer both *in vivo* and *in vitro*. Other work revealed MUC1 to be part of a miR-322-dependent regulatory loop in human carcinomas (33). In accordance with the present results, a previous study demonstrated that LincRNA-RoR/miR-145 accelerated the invasion and metastasis of triple negative breast cancer by up-regulating expression of MUC1 (34). Further, it has been suggested that miR-128 may serve as an attractive therapeutic strategy for paclitaxel-resistant lung cancer via inhibition of MUC1-C (35).

In conclusion, the present study demonstrated that overexpressing miR-497 through inhibiting methylation of its promoter region repressed proliferation and invasivity of breast cancer, and induced apoptosis in breast cancer cells through down-regulating MUC1 expression. Investigation of miR-497 and MUC1 in breast cancer cells and their functions yields a better understanding of their disease-related mechanisms and may present a promising new direction for the development of better treatments for breast cancer. However, the current study only presents the theoretical basis of this mechanism in breast cancer. Therefore, clinical experiments of fully developed miR-based anti-cancer therapeutic agents should be perfected in the future.

## Data Availability Statement

The original contributions presented in the study are included in the article/supplementary material, further inquiries can be directed to the corresponding author.

## Ethics Statement

The studies involving human participants were reviewed and approved by General Hospital of Ningxia Medical University. The patients/participants provided their written informed consent to participate in this study. The animal study was reviewed and approved by General Hospital of Ningxia Medical University.

## Author Contributions

ST, HL, and XM designed the study, and were involved in data collection. ST, HL, JH, and YG performed the statistical analysis and preparation of figures. XM and JL drafted the manuscript. BL checked the language usage of the whole manuscript. All authors contributed to the revision and approved the final manuscript.

## Conflict of Interest

The authors declare that the research was conducted in the absence of any commercial or financial relationships that could be construed as a potential conflict of interest.

## Footnotes

1 http://www.urogene.org/cgi-bin/methprimer/methprimer.cgi
